# The Model for Sustainable Mental Health: Future Directions for Integrating Positive Psychology Into Mental Health Care

**DOI:** 10.3389/fpsyg.2021.747999

**Published:** 2021-10-21

**Authors:** Ernst Bohlmeijer, Gerben Westerhof

**Affiliations:** ^1^University of Twente, Enschede, Netherlands; ^2^Center for eHealth and well-being, Enschede, Netherlands

**Keywords:** positive psychology, mental health care, intervention, implementation model, integration

## Abstract

This position paper proposes a model for systematic integration of positive psychology interventions (PPIs) in mental healthcare. On the one hand, PPIs can contribute to the decrease of dysfunctional processes underlying mental illness. This evidence is at the core of the new domains of positive clinical psychology and positive psychiatry. On the other hand, a growing number of studies demonstrate that mental health is not merely the absence of mental illness. Mental wellbeing represents a related but separate dimension of mental health. Mental wellbeing reduces the risk of future incidence of mental illness and is highly valued by people receiving psychological treatment as an important aspect of personal and complete recovery and personal growth. This makes mental wellbeing a vital outcome of mental healthcare. PPIs can directly increase mental wellbeing. The model of sustainable mental health is presented integrating the science of positive psychology and mental wellbeing into mental healthcare. This heuristic model can guide both practitioners and researchers in developing, implementing, and evaluating a more balanced, both complaint- and strength-oriented, treatment approach. The role of gratitude interventions is discussed as an example of applying the model. Also, three potential modalities for implementing PPIs as positive psychotherapy in treatment are as: positive psychotherapy as primary treatment, as combinatorial treatment, and as intervention for personal recovery of people with severe or persistent mental disorder. Finally, we argue that longitudinal studies are needed to substantiate the model and the processes involved.

## Introduction

Positive psychological interventions (PPIs) aim to enhance resources that enable people to be resilient and flourish. Some examples of evidence-based PPIs are: savoring, gratitude, kindness, optimism, promoting positive relationships, and pursuing meaning (Schueller and Parks, 2014). Though PPIs have been developed to promote happiness and optimal functioning in diverse groups like people who would like to grow and develop, children at school, workers in business (Seligman and Csikszentmihalyi, 2000; Linley et al., 2006), from the beginnings of positive psychology as a scientific domain, PPIs have been studied in clinical populations as well (Seligman et al., 2006). PPIs, aiming to promote positive affect, engagement, and social relationships, may improve response of psychological and psychiatric treatments of mental disorders which have led to initiatives to integrate positive psychology in clinical psychology (Wood and Tarrier, 2010; Wood and Johnston, 2016) and in psychiatry (Jeste and Palmer, 2015; Fava and Guidi, 2020).

In the past years, a rapidly growing number of studies have evaluated the effects of PPIs on mental health in clinical populations. Chakhssi et al. (2018) indeed found small to moderate effect sizes of PPIs on wellbeing, depression, and anxiety in clinical populations across 30 studies. In a recent comprehensive meta-analysis on the impact of PPIs, an average moderate effect on mental wellbeing was found, based on 60 studies in clinical populations (Carr et al., 2020). On the other hand, another recent meta-analysis has shown that a broad range of psychological interventions can contribute to the promotion of wellbeing in both clinical and non-clinical populations (van Agteren et al., 2021). In persons with mental illness, cognitive (behavioral) therapy had similar small to moderate effects on wellbeing as singular and multi-component PPIs.

Hence, an important question is how we can better understand the mutual effects of PPIs and more traditional psychological interventions on both mental wellbeing and mental illness. In this position paper, we present a heuristic model of sustainable mental health, systematically integrating PPIs in mental healthcare. We will first describe the model that is informed by empirical research but is mainly a theoretical proposition. Next, we use gratitude as an example of how it can be applied. We present three potential modalities for implementing PPIs as positive psychotherapy in treatment and close with directions for further research.

## Mental Wellbeing as a Vital Outcome of Mental Healthcare

The first step in developing the sustainable mental health model relates to the recognition that mental wellbeing is an important outcome in mental healthcare, besides mental illness. “Mental health is simply too important to be ignored” wrote eminent scholar George Vaillant (2003). The dominant focus in psychological treatment has long been on mental disorders and symptoms of psychopathology. Mental health, however, is more than the absence of mental illness. The World Health Organization accentuates the positive dimension of mental health defining it as “a state of well-being in which the individual realizes his or her own abilities, can cope with the normal stresses of life, can work productively and fruitfully, and is able to make a contribution to his or her community” (World Health Organization, 2005, p.2). The three core components of this definition are (1) feeling well, (2) effective functioning of an individual, and (3) effective functioning for a community.

In this definition, one can also recognize two traditions of wellbeing: one focusing on hedonic wellbeing and the other on eudaimonic wellbeing (Waterman, 1993; Ryan and Deci, 2001). There is widespread consensus that hedonic wellbeing is a multidimensional concept, including evaluations of life in emotional terms (i.e., life-satisfaction) as well as the presence of positive and absence of negative affect (Diener, 1984; Diener et al., 1999). The concept of eudaimonia dates back to Aristotle, for whom not subjective happiness, but the realization of one’s own potential in a societal context was the essential element of a good life (Waterman, 1993). Several models have been put forward for eudaimonic wellbeing, such as psychological wellbeing (Ryff, 2014) and self-determination theory (Ryan and Deci, 2017). Keyes (2005) developed a model of mental health in which emotional, psychological, and social wellbeing are all important indicators of a flourishing life.

Three key findings have emerged from studies on the relation between mental wellbeing and mental illness in the past decades. First, there is substantial evidence for the so-called two-continua model. This model holds that mental wellbeing and mental illness are related, yet discernible phenomena: One continuum represents the presence or absence of mental wellbeing and the other the presence or absence of mental illness. Superiority of a two-related factors model has been demonstrated in large representative surveys (Keyes, 2005; Westerhof and Keyes, 2010; Lamers et al., 2011; Weich et al., 2011; Schotanus-Dijkstra et al., 2017) as well as in clinical samples (Trompetter et al., 2017; De Vos et al., 2018; Franken et al., 2018). Evidence for mental wellbeing and mental illness as distinct dimensions also comes from, for example, health and life-style surveys (Huppert and Whittington, 2003) and life span studies (Westerhof and Keyes, 2010; Lamers et al., 2012).

Secondly, longitudinal evidence indicates that higher levels of mental wellbeing reduce the risk of incident mental health issues and mental disorders (e.g., Keyes et al., 2010; Wood and Joseph, 2010; Grant et al., 2013; Lamers et al., 2015; Schotanus-Dijkstra et al., 2017).

Thirdly, whereas the two-continua model has been developed from studies in the general population, studies on perspectives of mental healthcare users have made a difference between clinical and personal recovery (Slade, 2010). Based on a systematic review and narrative synthesis of qualitative studies on personal recovery, Leamy et al. (2011) developed the so-called CHIME model to describe five personal recovery processes: Connectedness, Hope and optimism, Identity, Meaning in life, and Empowerment. Although this model uses different terminology, there is a clear overlap with approaches to eudaimonic aspects of wellbeing. A systematic review and qualitative meta-analysis synthesized findings of qualitative studies on perspectives of persons who recovered from eating disorders in line with the two-continua model (de Vos et al., 2017). Besides a decrease in eating disorders-related behaviors and cognitions, they described improvements in self-acceptance, positive relationships, personal growth, self-adaptability/resilience, and autonomy as most fundamental criteria in their recovery process. Besides a reduction in complaints, these findings show that psychological wellbeing plays an important role in the recovery process for these persons. Hence, it can be concluded that the patient perspective also considers wellbeing an essential part of recovery processes.

Over the past years, many instruments have been developed to measure different aspects of mental wellbeing. An overview with as many as 99 instruments to measure mental wellbeing is available (Linton et al., 2010). Among the most often used are the Positive and Negative Affect Schedule, the Satisfaction with Life Scale, Ryff’s Scales of Psychological Well-being, the Warwick-Edinburg Mental Well-Being Scale, the Flourishing scale, and the Mental Health Continuum Short Form. A number of measures have also been developed for personal recovery (Van Weeghel et al., 2019), such as the Recovery Assessment Scale, the Stages of Recovery Instrument, and the Questionnaire on Personal Recovery. There is nowadays good evidence that people can provide valid and reliable self-reports of wellbeing (Diener et al., 2018).

In studies on the two-continua model, the Mental Health Continuum Short Form (Keyes et al., 2008) has become the most often used instrument. The instrument is solidly grounded in theoretical syntheses on subjective wellbeing and optimal psychological and social functioning with each item measuring a separate aspect of wellbeing. A recent meta-analytic structural equation model across 26 studies provided strong evidence of a general factor of mental wellbeing as well as three separate dimensions of emotional, psychological, and social wellbeing in both clinical and non-clinical samples (Iasiello et al., in press).

To summarize, growing evidence suggests that mental wellbeing is a vital outcome in mental healthcare that can be measured with reliable and valid instruments. Integrating PPIs in mental healthcare might contribute to a stronger focus on mental wellbeing in mental healthcare practice. However, PPIs are not meant to replace or compete with other well-established psychological treatments in mental healthcare but to complement them. But this raises the question how to optimally integrate PPIs in mental healthcare? To this end, we developed the model of sustainable mental health.

## The Model for Sustainable Mental Health

**Figure 1:**
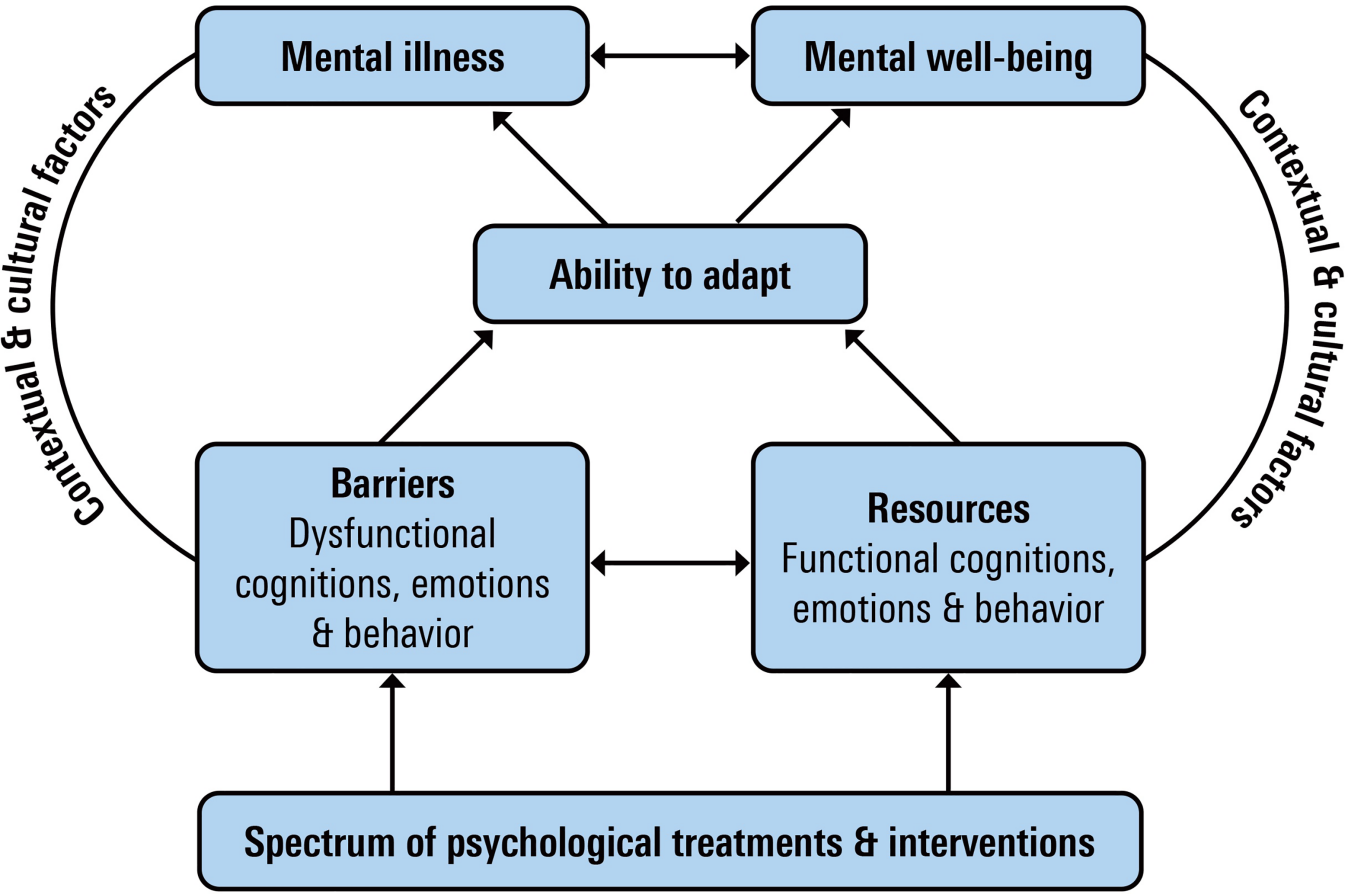
Model of sustainable mental health.

Figure 1 presents a model aiming to integrate dysfunctional and functional perspectives on mental health and psychological treatment. It is meant as a heuristic model guiding researchers and practitioners to develop, implement, and evaluate a more balanced, both complaints- and wellbeing-oriented, treatment approach. It is beyond the scope of this paper to discuss all components of the model in detail, but we give a brief overview of outcomes, adaptation, context, underlying processes, and interventions as main components of the model (Bohlmeijer and Westerhof, 2021).

A first component in this model is that sustainable mental health incorporates mental wellbeing as an essential mental health outcome besides mental illness as discussed in the previous section.

The second component refers to adaptation processes as inhibiting or promoting sustainable mental health. The absence of psychological complaints and the presence of mental wellbeing can easily be taken as normative endpoints. However, life is characterized by moments of suffering as well as flourishing. As both continua of mental health will fluctuate across time in a dynamic reciprocal process, it is important to better understand how people regulate their mental health, not in the least because that may also provide possibilities for psychological interventions. We therefore added adaptation processes to the model. Psychosocial adaptation can be seen as a *process* by which a person interacts with the psychosocial consequences of smaller or larger challenges in life. A common view is that temporality plays an important role in adaptation (Livneh, 2001). Short-term challenges include day-to-day moments that often involve emotions, such as anger, anxiety, or depression. These short-term reactions ask for coping processes that mainly focus on the reduction of stress (Lazarus and Folkman, 1984). Intermediate challenges may be referred to as self-management, which includes dealing with symptoms of illness, communication with health professionals, or adopting life-style changes (Barlow et al., 2002; Loriq and Holman, 2003). Lastly, there are longer term challenges that ask for assimilation and accommodation of life-goals, identity, and meaning in life. These processes are related to aspects of narrative identity development in which the way people identify with and distance themselves from difficult life experiences is key in the regulation of mental health and wellbeing (Westerhof and Bohlmeijer, 2012).

Besides the temporal aspect of adaptation processes, the regulatory focus of these processes might differ. A large body of psychological literature that is of interest to chart adaptation processes has focused on how to deal with stress, symptoms, and illnesses. These processes are mainly important in regulating the illness continuum of mental health, often with a reactive focus on solving problems, restoring previous levels of functioning, or bouncing back to an earlier equilibrium. More recently, a number of processes have been put forward in positive psychology that have a stronger focus on maintaining or promoting wellbeing, like the attention for positive emotions or the use of strengths, virtues, and values (Fredrickson, 2001; Lyubomirsky et al., 2005) and psychological flexibility (Kashdan and Rottenberg, 2010). Processes involved include, for example, benefit finding (Tennen and Affleck, 2005), post-traumatic growth (Tedeschi and Calhoun, 1995), and sense of coherence (Antonovsky, 1993). These processes focus more on the maintenance and promotion of wellbeing, despite the presence of problems and stress, or even on growth in wellbeing due to problematic or stressful experiences.

Thirdly, people may experience both barriers and resources for their adaptation processes. Many theories on mental illness as well as those on mental wellbeing distinguish between both in relationship to outcomes. Examples of well-studied barriers are information processing biases and negative biases in thinking, such as overgeneralization and magnification or minimization, dysfunctional schema and beliefs about the world and oneself, and suppression of emotions and avoidance behavior. Examples of resources are the ability to experience and amplify positive emotions, optimism, the awareness and use of strengths, values, self-affirmations, and positive relationships. We prefer the concepts of barriers and resources over, for example, negative and positive. The presence of negative emotions and cognitions, for example, can be functional in adaptation processes as they direct attention to important issues in the process. Over time, however, they may become dysfunctional when emotion regulation and coping strategies are inadequate and do not result in decreases of symptoms or increases in mental wellbeing.

Fourthly, contextual factors play an important role in supporting or thwarting individual attempts to maintain and achieve sustainable mental health. Ecological systems, such as relationships, parents, organizations, and communities, may either cause or maintain dysfunctional or maladaptive cognitions or behaviors that are related to mental illnesses or distress or may enhance resources that promote adaptation and mental health (Bronfenbrenner, 1995). It is important to acknowledge that the social, historical, and cultural context play an important role in supporting or thwarting individual attempts to maintain and achieve mental health. Although in this paper, we mainly focus on individual aspects of the model, this does not mean that individuals are solely responsible for managing their mental wellbeing and illness.

The fifth component is the spectrum of psychological treatments and interventions and their focus on targeting barriers or resources for successful adaptation. We propose that psychological treatments can be ordered on a spectrum, depending on whether their primary focus is on targeting barriers or resources for successful adaptation. At one end of the spectrum is psychological treatments or interventions that primarily target barriers for successful adaptation, e.g., CBT may target dysfunctional cognitions and avoidance behavior and schema therapy will target dysfunctional schema about the world and oneself. At the other end of the spectrum is psychological treatments or interventions, such as PPIs that primarily focus on developing and using resources for adaptation.

It is important to stress that many psychological treatments will comprise interventions targeting both barriers and resources for adaptation. One example is Acceptance and Commitment Therapy (ACT). ACT is a distinctive model of behavioral and cognitive therapy with a strong focus on the context of behavior (Hayes et al., 2013). It is based on a relational frame model that links behavioral principles of psychological flexibility to both pathology and flourishing (Ciarrochi and Kashdan, 2013; Hayes et al., 2013). ACT targets both barriers, such as experiential avoidance, and resources, such as awareness of and commitment to personal values. Hence, promoting sustainable mental health is a core focus in ACT (Bohlmeijer et al., 2015; Fledderus et al., 2015; White et al., 2017).

The model of sustainable mental health makes clear that there are several pathways to the different outcomes of mental illness and mental wellbeing. For example, an intervention may lead to fewer symptoms which in turn are related to more wellbeing. Or more specifically, an intervention might focus on strengthening resources that contribute to better adaptation and thereby to both mental wellbeing and mental illness. These kinds of processes might explain why PPIs can have an effect on mental illness (Chakhssi et al., 2018) as well as why CBT can have effects on wellbeing (van Agteren et al., 2021). However, it would be important to address the different barriers and resources as well as the aspects of adaptation and mental illness and mental wellbeing that are involved. We discuss these processes in some details regarding gratitude as an example.

## Gratitude

In this section, we want do discuss how gratitude as an example of a PPI fits into the model. Gratitude has been defined as both a positive affect resulting from the perception of receiving a benefit from another person (McCullough et al., 2002) and a trait, which includes the ability to appreciate simple things in life, sense of abundance and experience, and express gratitude toward others (McCullough et al., 2002; Watkins et al., 2003; Wood et al., 2010). People with gratitude as a stable character strength will also more frequently experience gratefulness as a positive emotion in daily life as they tend to notice and appreciate positive events and contributions of other people more often and more intensely (McCullough et al., 2002). A growing number of longitudinal studies have demonstrated the positive relation between gratitude and mental health (Wood et al., 2010). For example, it has been found that higher levels of gratitude predict improvements in wellbeing and distress over time for various populations, such as healthy adults (Disabato et al., 2017), heart patients (Millstein et al., 2016), people with rheumatic disorders (Sirois and Wood, 2017), and war veterans (Kashdan et al., 2006).

The most frequent studied gratitude interventions are the gratitude journal and gratitude letters (Emmons, 2013). The gratitude journal intervention invites people to write every day for 10 or 15min about events and experiences they are grateful for. In the gratitude letter intervention, people write a letter to person expressing their gratitude explaining how the other person has had a positive impact on one’s life. People are then invited to visit the other person and read the letter out aloud. In both interventions, writing in detail about the experienced benefits is a key working mechanism of gratitude. Reviews found limited and inconclusive evidence for the efficacy of mainly brief gratitude interventions (Wood et al., 2010; Davis, 2016; Dickens, 2017). However, recent studies showed a substantial impact on mental health of gratitude interventions of longer duration (Heckendorff et al., 2018; Bohlmeijer et al., 2021).

**Figure 2:**
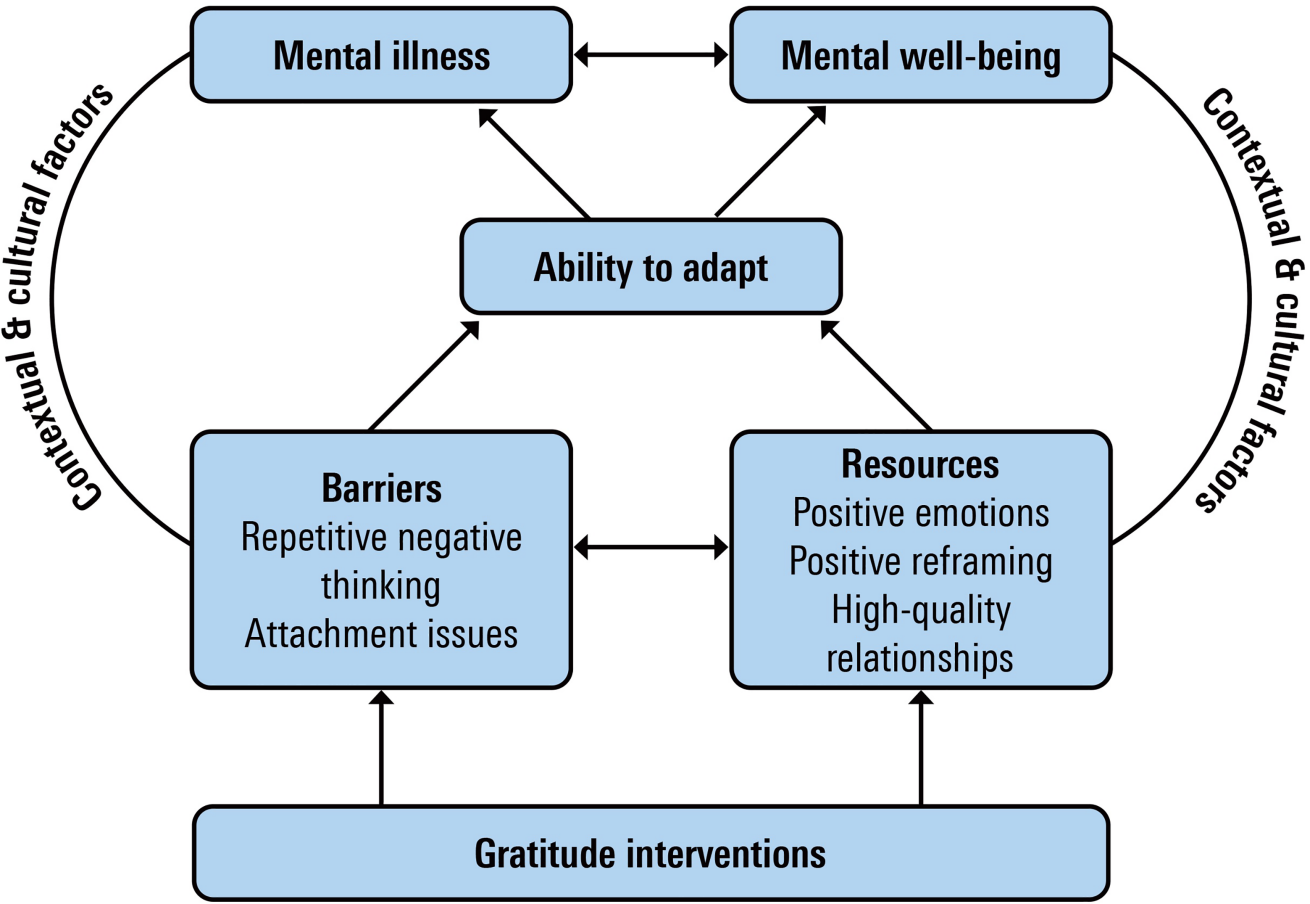
Gratitude and model of sustainable mental health.

Figure 2 shows the potential impact of gratitude interventions within the model of sustainable mental health.

First of all, gratitude interventions may build *resources* that promote adaptation and contribute to mental health. For example, gratitude interventions aim to enhance the daily experience of gratefulness as a positive emotion. The broaden-and-build theory (Fredrickson, 2001) demonstrates how gratitude and other positive emotions contribute to a more broadened thought-action repertoire (Fredrickson, 2004a). This broadened repertoire sets into motion two positive spirals. First, it will promote the likelihood of discovering meaning in upcoming events, increasing the experience of positive affect in the near future (Fredrickson and Joiner, 2002). Secondly, over time positive emotions cause a positive spiral building durable physical, cognitive, and social resources promoting the ability to adapt and mental health (Fredrickson, 2003). Thus, gratitude as a life-orientation will directly contribute to mental wellbeing by a tendency toward noticing and appreciating the positive in the world and gratitude as a positive affect will decrease distress by building mental flexibility (Wood et al., 2010). Longitudinal studies have found a relationship between gratitude and negative mental states, such as depression mediated by positive emotions (e.g., Lambert et al., 2012). Also, gratitude interventions may promote the use of adaptive coping-styles, such as positive reframing. Positive reframing is the ability to develop positive interpretations of events that are firstly experienced as negative. An example is when an adverse life-event is considered as an opportunity to deepen a relation or develop new skills. Several studies have found evidence for the mediational role of positive reframing in the relationship between gratitude and mental health (Lambert et al., 2009, 2012). Watkins et al. (2008) found that grateful processing was beneficial in bringing closure to negative emotional memories. Finally, there is also ample evidence demonstrating that experiencing and expressing gratefulness supports the development and maintenance of high-quality relationships (e.g., Algoe et al., 2010; Grant and Gino, 2010; Lambert and Fincham, 2011; Algoe and Zhaoyang, 2016). Experiencing gratitude draws our attention to people who, by their small or major gesture, demonstrate that they care for us and are responsive to us (Algoe et al., 2013). By expressing appreciation, we confirm emotional closeness with the other person. Receiving appreciated benefits and expressing gratitude promotes shared joy and reciprocal kindness, relationship satisfaction, and willingness to invest in the relationship, all key positive, interpersonal processes (Algoe, 2019). High-quality relationships have been found to play a vital role in mental health (e.g., Thoits, 2011).

Secondly, gratitude interventions may impact *barriers* for adaptation. One such barrier is repetitive negative thinking. This is considered an important transdiagnostic risk factor for psychopathology (Spinhoven et al., 2015) comprising sustained focus on and difficulty to disengage from negative content. Gratefulness promotes noticing and appreciating positive experiences and this may train people to shift their attentional focus from negative experiences to more positive ones. Several studies have found evidence for the beneficial effects of gratitude interventions on repetitive negative thinking (Heckendorf et al., 2019; Wood and Hyland, 2010). However, the relation between gratitude and barriers may also work the other way around. One example is the presence of a dysfunctional attachment style. People who score high on attachment-related anxiety have difficulties in trusting that a partner will be responsive in times of need, and people with an attachment-avoidance profile are more distrustful of the goodwill of other people (Brennan et al., 1998; Mikulincer and Shaver, 2009). People with strong attachment issues will have difficulties in noticing and appreciating good intentions and deeds of other people and in expressing gratefulness. Thus, the presence of attachment issues may inform the practitioner not to indicate gratitude as an intervention.

## Combining Positive Psychotherapy with Complaint-Oriented Treatments

In the last section, we discussed gratitude as an example of one positive psychology intervention. In the context of psychological treatment, several positive psychology inventions are often combined into so-called positive psychotherapy. Positive psychotherapy can be defined as a treatment approach that systematically amplifies the positive resources of clients, such as strengths, meaning, positive relationships, and intrinsically motivated accomplishment with aim of increasing mental wellbeing and personal recovery and undoing or diminishing mental illness (Rashid and Howes, 2016). How can positive psychotherapy be combined with treatments and interventions primarily aiming to reduce barriers for adaptation (complaint-oriented psychotherapy)? We discuss three potential modalities: positive psychotherapy as primary treatment, as a combinatorial treatment, and as personal recovery treatment.

Positive psychotherapy can be offered as *primary treatment*. Chaves et al., 2017 found that the effects of positive psychotherapy in reducing depressive symptomatology were similar to the effects of cognitive behavioral therapy (CBT) in a sample of women with a DSM-IV diagnosis of major depression. One explanation is that a lack of positive emotions and cognitions plays a major role in causing and maintaining depression (Gotlib and Joormann, 2010; Watson and Naragon-Gainey, 2010; Carl et al., 2013) and positive psychotherapy is primarily aimed at increasing positive emotions and cognitions. Though evidence-based treatments for depression, such as CBT, have been established (Cuijpers et al., 2011), it has also been estimated that about 10–60% of patients with depressive disorders do not respond to evidence-based treatments (Carvalho and Mcintyre, 2015). Moreover, about 20% of patients with a depressive disorder develop a chronic or therapy-resistant course (Kerkhof, 2002; Spijker et al., 2002). Possibly some patients may respond better to positive psychotherapy. Analyses using the Personalized Advantage Index showed that patients mental and physical comorbidity, prior antidepressant medication, higher levels of negative cognitions, and higher personal growth demonstrated a better response to positive psychotherapy in comparison with CBT (Lopez-Gomez et al., 2019). However, more research on factors predicting response to specific treatments is warranted.

Implementing positive psychotherapy as a *combinatorial treatment* is a second modality. One example is sequential treatment that can be defined as a two-stage approach, based on the *a priori* assumption that one treatment will often not be successful in realizing complete recovery or full response (Guidi et al., 2016). For example, studies have found larger effects on mental health for sequential combinations of CBT and wellbeing therapy in comparison with active control groups for people with cyclothymic disorder (Fava et al., 2011) and people with acute coronary syndromes (Rafanelli et al., 2020). Though wellbeing therapy differs from positive psychotherapy, these treatments share a primary focus on increasing psychological wellbeing. Within the model of sustainable mental health, sequential treatment may imply that a treatment primarily targeting barriers for adaptation is followed by a treatment primarily focusing on increasing resources for adaptation. A second example is implementing positive psychotherapy as a relapse prevention or rehabilitation intervention. Radstaak et al. (2020) evaluated the impact of an intervention combining wellbeing therapy and positive psychology as a rehabilitation intervention for people who had been treated for PTSD. For patients with lower levels of wellbeing, indicating the absence of complete recovery, the positive intervention was more effective than treatment as usual for mental wellbeing and post-traumatic growth.

A third modality is implementing positive psychotherapy as an intervention for personal recovery and wellbeing. This is especially relevant for people with severe mental illness (SMI) that can be defined a psychiatric disorder with severe functional problems, where the constraints are causal and consequential and which is not temporary, and there is a need for coordinated professional care (Delespaul, 2013). Examples of SMIs based on these criteria are schizophrenia, schizoaffective disorder, bipolar disorder (BD), personality disorder, and major depressive disorders (MDD). Several positive psychotherapy programs in this context have been developed. For example, WELLFOCUS is a positive psychotherapy group program adapted for people with psychosis. A trial with 94 participants showed no significant effects for mental wellbeing but small to moderate effects for various symptoms of mental ill-being in comparison with treatment as usual (Schrank et al., 2016). Another example is the *Living well with mental disorder* intervention (Kraiss et al., 2018). This is an 8-week transdiagnostic positive psychotherapy group intervention for people with SMI. Kraiss et al. (2018) conducted a trial on the effects of this intervention in a sample of 97 patients with bipolar disorder. The post-treatment findings show a moderate to large effect on mental wellbeing and personal recovery in comparison with treatment as usual (Kraiss, 2021). Recently, a meta-analysis on the effects of PPIs on mental health in people with SMI across studies was conducted (Geerling et al., 2020). Sixteen studies were included (nine RCTs), representing 729 patients. The results showed that there is at present no evidence that PPIs are more effective in comparison with other active interventions. However, within-group effects revealed a moderate effect (Hedge’s *g*=0.40) on wellbeing and a large effect on mental ill-being symptomatology (*g*=0.70), suggesting that people with SMI do benefit from PPIs in terms of enhancement of mental health.

## Implications for Future Research

In the last section, we discussed gratitude as an example of one PPI and tried to explain the pathways through which it can have effects on both mental wellbeing and mental illness as outcomes. Overall, it has been discussed that it is difficult to demonstrate the superiority of one intervention type over another as most psychological interventions have some kind of effect on mental illness (Lambert, 2013a; Lambert, 2013b). This might apply to effects on mental wellbeing as well. Hence, it would be of value to conduct similar exercises for other kinds of interventions, thereby filling the model with different resources, aspects of adaptation, and pathways to outcomes. This could help to better understand what the main outcomes and working mechanisms of different forms of interventions in clinical samples are.

Although the model is informed by empirical research, it is mainly a theoretical proposition. A next step would be to further substantiate the model with empirical studies. This would provide more insight into the validity of the heuristic model for specific PPIs. Structural equation modeling could be used to examine the validity of the model. Researchers would need to make good choices for instruments to measure the different components in the model, but they would also need to have good longitudinal designs to assess the processes involved. Intervention studies assessing the impact of PPIs on mental health could include measures of adaptation, barriers, and resources and mediational analyses could study the specific processes of change as predicted by the model of sustainable mental health. This would also include research on the different modalities of combining PPIs and other psychological interventions. Besides demonstrating that interventions do have an effect on mental health outcomes, this would therefore also involve research that gives a better understanding what works when for whom.

This research agenda would also entail innovations in research methods. For example, single-case designs with more measures for fewer participants can be used as they are rather flexible in tailoring research to the processes that are involved in specific interventions. Furthermore, the APA describes including professional expertise and patient perspectives as important besides scientific evidence for effectiveness. These perspectives can add to filling in the model for particular interventions, but they can also inform theory on good, shared decision making about modalities and what works when for whom.

The model for sustainable mental health we presented in this position paper primarily applies to clinical psychology and psychiatry and not to public mental health and prevention. We also realize that the model is most relevant to western countries with a developed capacity for mental healthcare. It has been shown that most positive psychology research has been conducted in western, educated, industrialized, rich, and democratic (WEIRD) samples (Hendriks et al., 2018). It is also important to stress that wellbeing is strongly influenced by social and cultural contexts (McNulty and Fincham, 2011). For example, wellbeing literacy has been introduced as a vital capability to comprehend and compose wellbeing language across contexts in order to improve wellbeing of self and others (Oades et al., 2020, 2021). Though the model for sustainable mental health acknowledges the relevance of contextual and cultural factors mental health, it was beyond the scope of this paper to discuss them.

## Author Contributions

All authors have written and equally contributed to the paper.

## Conflict of Interest

The authors declare that the research was conducted in the absence of any commercial or financial relationships that could be construed as a potential conflict of interest.

## Publisher’s Note

All claims expressed in this article are solely those of the authors and do not necessarily represent those of their affiliated organizations, or those of the publisher, the editors and the reviewers. Any product that may be evaluated in this article, or claim that may be made by its manufacturer, is not guaranteed or endorsed by the publisher.
